# Direction of Arrival Estimation of Coherent Wideband Sources Using Nested Array

**DOI:** 10.3390/s23156984

**Published:** 2023-08-06

**Authors:** Yawei Tang, Weiming Deng, Jianfeng Li, Xiaofei Zhang

**Affiliations:** College of Electronic and Information Engineering, Nanjing University of Aeronautics and Astronautics, Nanjing 211106, China; ywtang@nuaa.edu.cn (Y.T.); dengweiming0202@126.com (W.D.); zhangxiaofei@nuaa.edu.cn (X.Z.)

**Keywords:** direction-of-arrival (DOA), sparse array, initial-estimation-free CSSM, enhanced spatial smoothing

## Abstract

Due to their ability to achieve higher DOA estimation accuracy and larger degrees of freedom (DOF) using a fixed number of antennas, sparse arrays, etc., nested and coprime arrays have attracted a lot of attention in relation to research into direction of arrival (DOA) estimation. However, the usage of the sparse array is based on the assumption that the signals are independent of each other, which is hard to guarantee in practice due to the complex propagation environment. To address the challenge of sparse arrays struggling to handle coherent wideband signals, we propose the following method. Firstly, we exploit the coherent signal subspace method (CSSM) to focus the wideband signals on the reference frequency and assist in the decorrelation process, which can be implemented without any pre-estimations. Then, we virtualize the covariance matrix of sparse array due to the decorrelation operation. Next, an enhanced spatial smoothing algorithm is applied to make full use of the information available in the data covariance matrix, as well as to improve the decorrelation effect, after which stage the multiple signal classification (MUSIC) algorithm is used to obtain DOA estimations. In the simulation, with reference to the root mean square error (RMSE) that varies in tandem with the signal-to-noise ratio (SNR), the algorithm achieves satisfactory results compared to other state-of-the-art algorithms, including sparse arrays using the traditional incoherent signal subspace method (ISSM), the coherent signal subspace method (CSSM), spatial smoothing algorithms, etc. Furthermore, the proposed method is also validated via real data tests, and the error value is only 0.2 degrees in real data tests, which is lower than those of the other methods in real data tests.

## 1. Introduction

In recent years, DOA estimations [1,2,3] have been widely applied in many fields, such as radar, sonar, wireless communication [4,5,6,7], etc. Therefore, research into DOA estimation has attracted more attention from researchers [8,9,10,11,12]. In general, the spectral estimation accuracy is positively correlated with the number of antennas, while the sparse arrays can obtain a large number of continuous elements in the virtual array via the virtualization method, thereby obtaining a higher accuracy of DOA estimation. To improve the accuracy of DOA estimation and increase the DOF with a fixed number of antennas, sparse arrays have gradually replaced uniform line arrays as the most prevalent array geometry. Examples of these arrays include the minimum redundancy array (MRA) [13], the coprime array and the nested array. Compared with MRA, coprime array [14,15,16] and nested array [17,18] are more feasible in terms of engineering and they can obtain a large number of continuous elements in the virtual array via the virtualization method, thereby obtaining a higher accuracy of DOA estimation and a larger DOF. However, nowadays, research based on sparse arrays is mainly used for incoherent signals, as the performance is poor at handling coherent wideband signals. In reality, it can be challenging to evade coherent signals. As a result, the resolution of coherent wideband signals in sparse arrays is the primary focus of this paper.

Currently, incoherent narrowband signal DOA estimation techniques have been extensively used. However, due to the complex propagation environment [19,20], coherent wideband signals have become an important signal type in real life, and the coherent signal becomes an important factor affecting the performance of the algorithm. Traditional incoherent narrowband DOA estimation methods are inadequate for handling coherent wideband signals. Therefore, research into DOA estimation methods for coherent wideband signals holds significant practical importance. Many algorithms have been developed to counteract the effect of coherence [21,22,23,24,25,26], such as the widely used algorithm known as spatial smoothing pre-processing (SSP) [27], which divides the entire array into a series of overlapping subarrays to obtain a new data covariance matrix with recovered rank. In this paper, in order to take full advantage of the covariance matrix of individual subarrays and the mutual covariance matrix of different subarrays, an enhanced spatial smoothing algorithm is used to more effectively counteract the effects of coherence.

For wideband signals, many types of studies have been conducted regarding wideband DOA estimation algorithms. Usually, the wideband signal is first decomposed into several narrowband signals with multiple frequencies via time-to-frequency conversion. Then, two widely used classes of wideband DOA estimation algorithms are used. One is the incoherent signal subspace method (ISSM) [28,29,30], which applies a high-precision narrowband DOA estimation algorithm (such as the MUSIC [31] algorithm) to the narrowband signals at multiple frequencies after the time–frequency conversion and averages the results to obtain the estimated values. This method can achieve high accuracy in a high SNR, but in a low SNR, the accuracy of the algorithm may be greatly affected, and this method cannot effectively deal with the problem of coherent sources [32]. Moreover, another algorithm, known as the coherent signal subspace method (CSSM) [33,34,35,36,37], is often proposed, which focuses the signal subspace at multiple frequencies to the signal subspace at the reference frequency by constructing a focus matrix and summing these focused covariance matrices to construct a single correlation matrix, after which a high-precision narrowband DOA estimation algorithm can be applied to this covariance matrix to obtain the final estimated value. This algorithm achieves great performance in the case of low SNR, and the process of averaging after focusing can reduce the coherence coefficient between signals. Therefore, it can achieve some decorrelation effect. However, the construction of the focusing matrix in this algorithm depends on the pre-estimated angle, and the accuracy of the pre-estimated angle will have a large impact on the accuracy of the final estimated angle. Therefore, a focusing algorithm that does not require pre-estimated values is used in this paper to avoid the main drawbacks of the traditional focusing algorithm.

In this paper, firstly, we choose a sparse array in order to obtain a higher spectral estimation accuracy and a larger DOF using a fixed number of antennas. Then, we choose the CSSM method to assist in the process of decorrelating the coherent signal. In order to avoid the final estimation result relying on the pre-estimation, we propose a focusing algorithm without pre-estimation. Then, we virtualize the covariance matrix of sparse array due to the decorrelation operation. To more effectively counteract the effects of coherence, we adopt an enhanced spatial smoothing algorithm to make full use of the information in the covariance matrix of individual subarrays and the mutual covariance matrix of different subarrays. Finally, the MUSIC algorithm is applied to obtain the final estimated values.

The paper is organized as follows: In the Section 2, the sparse array based wideband coherent signal model is described. Then, the algorithm proposed in this paper is presented in the Section 3. The simulations, actual measurement and analysis are mentioned in the Section 4. Finally, the conclusion is presented in the Section 5 of this paper.

## 2. Array Model

**Figure 1:**
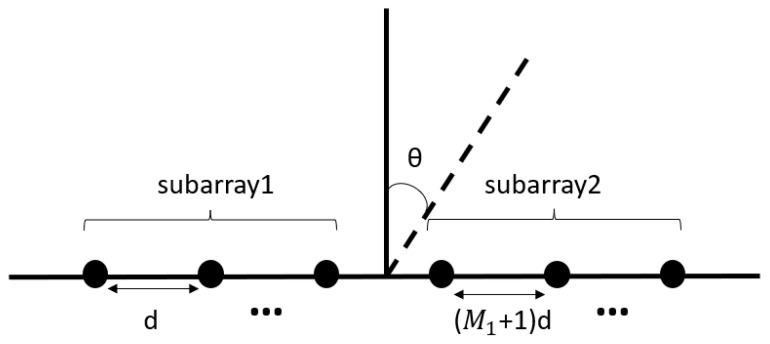
Two-level nested array.

Considering that the K far-field wideband sources impinged on a two-level nested array, the received signals were coherent. The sensor positions of nested array could be expressed as $P=\{m_{1}d,1\leq m_{1}\leq M_{1}\}\cup \{m_{2}(M_{1}+1)d,1\leq m_{2}\leq M_{2}\}$, where d=λ/2 (λ denotes the signal wavelength), and $M_{1}$ and $M_{2}$ represented the number of sensors of the each of the subarrays, the array is shown in Figure 1. The wideband sources from different DOAs $\theta_{1},\theta_{2},\cdots ,\theta_{k}$ were assumed to be independent. The array output of the m−th sensor could be represented as follows:

(1) $$x_{m}(t)=\sum_{k=1}^{K}s_{k}(t-\tau_{km})+n_{m}(t),m=1,2,\cdots ,M_{1},M_{1}+1,2(M_{1}+1),\cdots M_{2}(M_{1}+1)$$

where $s_{k}(t)$ and $n_{m}(t)$ represent the k−th source signal and the noise at the m−th sensor, respectively, and $n_{m}(t)$ is the additive white Gaussian noise with zero mean and variance $\sigma^{2}$. $\tau_{km}$ is the propagation delay associated with the k−th source and m−th sensor.

(2) $$\tau_{km}=(m-1)d\sin\theta_{k}/c$$

where c is the speed of signal propagation.

We transformed the output of each sensor into the frequency domain and partitioned the frequency band into J non-overlapping narrowband blocks via J−point DFT. Then, the wideband array model at J frequencies can be formulated as follows:

(3) $$X(f_{j})=A(f_{j})S(f_{j})+N(f_{j}),\;\;j=1,2,\cdots ,J$$

where $S(f_{j})={\left[S_{1}(f_{j}),S_{2}(f_{j}),\cdots ,S_{K}(f_{j})\right]}^{T}$ and $N(f_{j})={\left[N_{1}(f_{j}),N_{2}(f_{j}),\cdots ,N_{M}(f_{j})\right]}^{T}$ represent the signal and noise at frequency $f_{j}$ after DFT transformation, respectively.

(4) $$A(f_{j})=[a_{\theta_{1}}(f_{j}),a_{\theta_{2}}(f_{j}),\cdots a_{\theta_{K}}(f_{j})],\;\;j=1,2\cdots J$$

where $a_{\theta_{k}}(f_{j})={\left[e^{-j2\pi f_{j}\tau_{k1}},e^{-j2\pi f_{j}\tau_{k2}},\cdots ,e^{-j2\pi f_{j}\tau_{kM}}\right]}^{T}$ represents the frequency-dependent array steering vectors for k−th source angles.

The data covariance matrix can be written as follows:

(5) $$\begin{array}{ll} R(f_{j}) & =E[X(f_{j})X^{H}(f_{j})]\\ & =A(f_{j})Rss(f_{j})A^{H}(f_{j})+\sigma^{2}I \end{array}$$

where $Rss(f_{j})=E[S(f_{j})S^{H}(f_{j})]$ is the covariance matrix of the source signals at frequency $f_{j}$.

## 3. Proposed Method

### 3.1. Multi-Frequency Focused Decorrelation Method

The traditional CSSM algorithm was used to focus information derived from different frequency bins on a reference frequency bin, and narrowband algorithms were then used to obtain DOA estimation values. The key purpose of this algorithm was to construct a focusing matrix $T(f_{j})$ to transform the array manifold at frequency $f_{j}$ to the reference frequency $f_{0}$ as follows:

(6) $$T(f_{j})A(f_{j})=A(f_{0})$$

where $T(f_{j})$ is the focusing matrix at frequency $f_{j}$, and it is a unitary matrix.

The data covariance matrix was generated as follows:

(7) $$R_{c}=\sum_{j=1}^{J}T(f_{j})R(f_{j})T^{H}(f_{j})$$

The focusing matrices at different frequencies are non-unique, and the methods used to construct this matrix have been extensively explored. The rotational signal subspace (RSS) algorithm is now the approach most widely used to construct unitary focusing matrices by minimizing the Frobenius norm of the array manifold mismatches.

However, for most existing methods of constructing focus matrices, initial estimations were critical, and poor initial estimations could have a significant impact on the final DOA estimations.

In order to avoid excessive dependence on initial estimations for the final accuracy, we adopted a focusing algorithm that did not require pre-estimation. It can be observed that the unitary matrix with columns that are the eigenvectors of the data covariance matrix spanned the same subspace across each frequency as the array manifold, meaning that we can use them to construct the focusing matrix. This method does not require initial estimations compared to the approach of building a focusing matrix based on the array steering vector.

Firstly, we define the focusing matrix as follows:

(8) $$T_{auto}(f_{j})=\frac{1}{\sqrt{J}}U(f_{0})U^{H}(f_{j})$$

where $U(f_{j})$ is a unitary matrix, and its columns are the eigenvectors of the covariance matrix $R(f_{j})$.

In practical cases, we multiply $T_{auto}(f_{j})$ and $X(f_{j})$, and we then find

(9) $$Y(f_{j})=T_{auto}(f_{j})X(f_{j})$$

The covariance matrix can be presented as follows:

(10) $$\begin{array}{ll} R_{y}(f_{j}) & =E[Y(f_{j})Y^{H}(f_{j})]\\ & =T_{auto}(f_{j})A(f_{j})Rss(f_{j})A^{H}(f_{j})T_{auto}{}^{H}(f_{j})+\frac{1}{J}\sigma^{2}I \end{array}$$

Summing up the matrices at J frequencies, we can write the covariance matrix as follows:

(11) $$\begin{array}{ll} R_{coh} & =\sum_{j=0}^{J-1}R_{y}(f_{j})\\ & =\frac{1}{J}U(f_{0})\left(\sum_{j=0}^{J-1}U^{H}(f_{j})A(f_{j})Rss(f_{j})A^{H}(f_{j})U(f_{j})\right)U^{H}(f_{0})\\ & +\sigma^{2}I \end{array}$$

By constructing a focusing matrix to focus all frequency components to the reference frequency and then averaging the covariance matrix, the correlation coefficients between the signals were reduced, meaning that the rank of the covariance matrix was equal to the number of sources used for decorrelation.

Finally, we constructed the focusing matrix without the need for initial estimations, focused the covariance matrices at different frequencies to the reference frequency $f_{0}$, and, ultimately, obtained the required covariance matrix.

Since the array we used was a two-level nested array, it needed to be virtualized before it could be used to perform DOA estimation. Thus, we vectorized the above covariance matrix and found

(12) $$z=vec(R_{coh})$$

We sorted z and removed redundancy to obtain $\tilde{z}$, which is the received signal of this virtual array. The number of continuous elements in the virtual array is $N=2M_{1}(M_{2}+1)-1$.

### 3.2. Enhanced Spatial Smoothing Method

**Figure 2:**
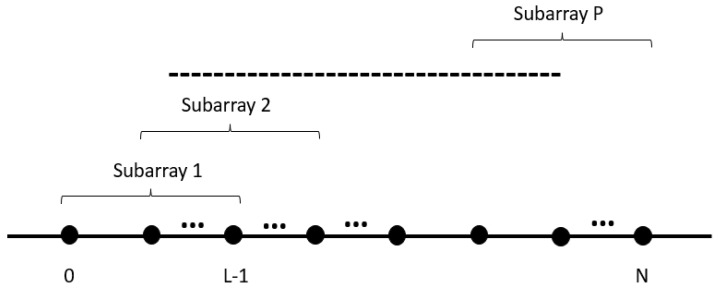
Overlapping subarrays used in the spatial smoothing method.

As the received signal is coherent, subspace-based and propagator-based algorithms cannot be used directly. And one of the most famous decorrelation algorithms is the spatial smoothing algorithm. The entire array is divided into P subarrays, and L represents the number of array elements in each subarray, the subarray is shown in Figure 2, where dotted line represents subarrays not shown in the figure. The relationships between P, L and N can be expressed as follows:

(13) N=L+P−1

Therefore, the cross-covariance matrix $R_{ij}$ of the i−th subarray and the j−th subarray can be written as follows:

(14) $$R_{ij}=E[\tilde{z_{i}}{\tilde{z_{j}}}^{H}]\;\;\;\;i,j\in (1,2,\cdots P)$$

where $\tilde{z_{p}}$ represents the p−th L row of $\tilde{z}$.

And the backward cross-covariance matrix can be expressed as follows:

(15) $$\bar{R_{ij}}=JR_{ij}^{*}J$$

where J denotes the (L×L) exchange matrix, and the operator * represents the complex conjugate.

One of the drawbacks of many spatial smoothing methods is that they do not make full use of the information in the subspace, while the spatial smoothing method used in this paper utilizes information including the cross-covariance matrix $R_{ij}$ of different subarrays and the covariance matrix $R_{ii}/R_{jj}$ of a single subarray. The rank-restored data covariance matrix after enhanced spatial smoothing can be written as follows:

(16) $$\begin{array}{ll} R_{ESS} & =\frac{1}{2P}\sum_{i=1}^{P}\sum_{j=1}^{P}\{\left(R_{ij}R_{ji}+\bar{R_{ij}}\bar{R_{ji}}\right)\\ & +\left(R_{ii}R_{jj}+\bar{R_{ii}}\bar{R_{jj}}\right)\} \end{array}$$

Furthermore, we apply the MUSIC algorithm to the covariance matrix $R_{ESS}$, and the covariance matrix $R_{ESS}$ can be divided into the signal subspace and the noise subspace, which can be written as follows:

(17) $$R_{ESS}=U_{s}\Sigma_{s}U_{s}^{H}+U_{N}\Sigma_{N}U_{N}^{H}$$

where $\Sigma_{s}$ is the diagonal matrix consisting of the maximum eigenvalues sorted in descending order, which have the same numbers as the sources. $\Sigma_{N}$ is the diagonal matrix composed of the other eigenvalues. $U_{s}$ and $U_{N}$ are matrices with the eigenvectors that correspond to the eigenvalues as columns.

The MUSIC spectrum is generated as follows:

(18) $$P_{\mathrm{MUSIC}}(\theta )=\frac{1}{a_{1}^{H}U_{N}U_{N}^{H}a_{1}}$$

where $a_{1}={\left[0,e^{-j2\pi f_{0}d\sin\theta /c},e^{-j2\pi f_{0}(2d)\sin\theta /c},\cdots ,e^{-j2\pi f_{0}(L-1)d\sin\theta /c}\right]}^{T}$ is the subarray steering vector at the reference frequency $f_{0}$.

Then, DOA estimation values are obtained through a spectrum search. The main steps of the proposed method are shown in Algorithm 1.
**Algorithm 1:** The Proposed MethodInput: The received data $X(f_{j})$ Output: DOA estimation valuesConstruct the focus matrix $T_{auto}(f_{j})$ according to Equation (8);Obtain the covariance matrix $R_{coh}$ after focusing according to Equations (10) and (11);Vectorize the above covariance matrix $R_{coh}$ and obtain the received signal $\tilde{z}$ of the virtual array according to Equation (12);Apply an enhanced spatial smoothing method to obtain the rank-restored covariance matrix $R_{ESS}$ according to Equations (14)–(16); Apply the MUSIC algorithm to obtain the estimation results according to Equations (17) and (18)


## 4. Performance Analysis

### 4.1. Simulation Analysis

To prove the superiority of the proposed method, we compared it to several other methods. The four methods used are as follows: (i) Using the method in [15], which uses conventional ISSM algorithm, decorrelation was performed via the traditional spatial smoothing algorithm (named ISSM-ss); (ii) after constructing the focus matrix using the traditional CSSM algorithm, we used the enhanced spatial smoothing algorithm for decorrelation (named CSSM-enss); (iii) we then used the focusing algorithm that does not require the initial estimation to generate the focus matrix (named IEF-CSSM); and (iv) the algorithm proposed in this article was used.

In this section, the root mean square error (RMSE) was used to verify algorithm performance. The expression of RMSE is as follows:

(19) $$\mathrm{RMSE}=\sqrt{\frac{1}{KN}\left(\sum_{n=1}^{N}\sum_{k=1}^{K}{\left(\theta_{k}-{\hat{\theta }}_{k,n}\right)}^{2}\right)}$$

where K and N denote the numbers of sources and Monte Carlo trials, respectively. ${\hat{\theta }}_{k,n}$ represents the estimated values of n−th Monte Carlo trial. In the following simulations, the number of Monte Carlo trials is 500.

We design two wideband sources impinging on a two-level nested array from 20° and 45°, and the number of sensors used for both subarrays is 10. The more frequency bins there are present, the slower the processing speed will be, and the accuracy of the results will raise. When the number of frequency bins reaches a certain threshold, the change in result accuracy will become negligible or even non-existent. As a result, between 280 and 320 MHz, a total of 40 frequency bins is uniformly sampled for wideband DOA estimation.

**Figure 3:**
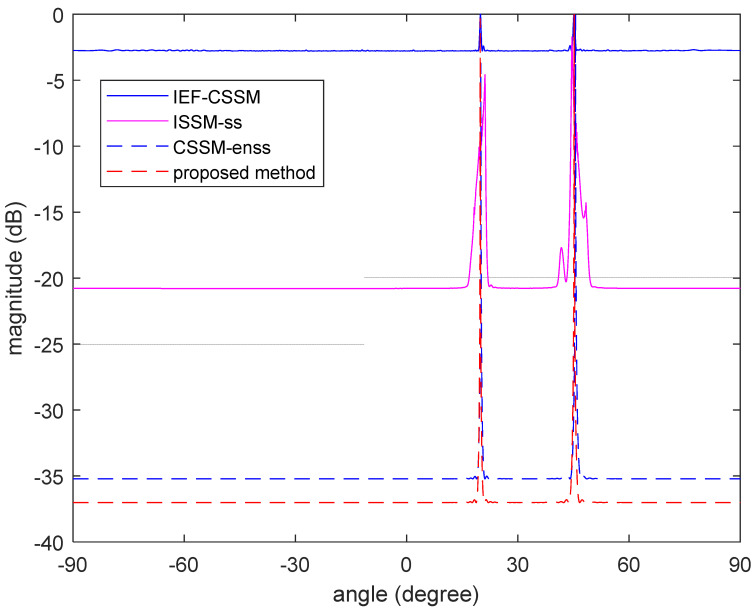
Comparison between the spatial spectra of different algorithms.

As shown in Figure 3, all four methods perform decorrelation and have spectral peaks present around the angle of the two sources, but the heights of the spectrum peaks are different due to the varying performances of the decorrelation methods. The method with the lowest peak is the IEF-CSSM method, which proves that the CSSM method is indeed helpful in decorrelation, while the method with a lower peak is the ISSM-ss method. There are some deviations in the positions of the spectral peaks due to the use of the ISSM method and the common spatial smoothing algorithm, which is not as effective as the other two methods using the enhanced spatial smoothing algorithm. Moreover, the CSSM-enss method results in the final spectral peak height being lower than that of the method proposed in this paper due to the difference in the focusing algorithm.

**Figure 4:**
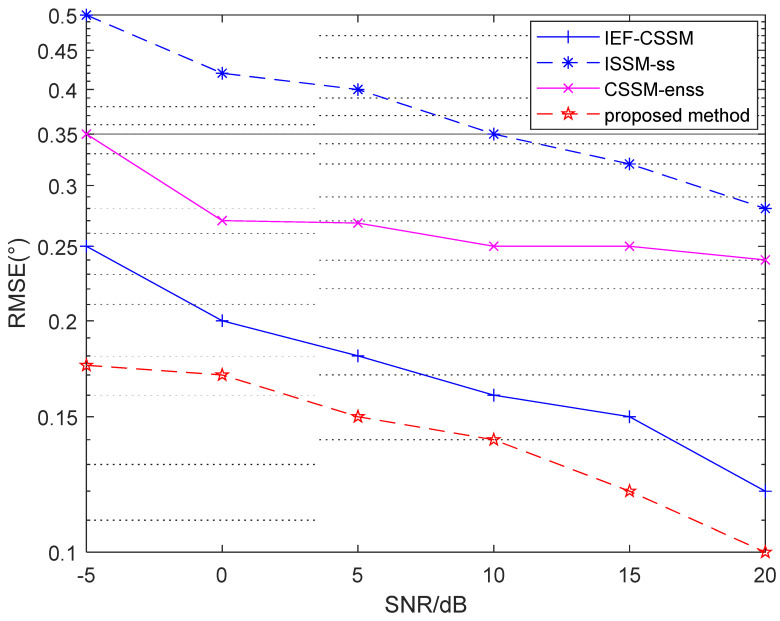
RMSE versus SNR.

As shown in Figure 4, the RMSE of each method becomes smaller as the SNR gradually increases. Among the methods, the CSSM-enss method suffers from a definite impact on the final estimates because the method used to construct the focus matrix is the conventional CSSM algorithm that requires an initial estimation value, though CSSM-enss has a better performance than ISSM-ss in its decorrelation algorithm, meaning that the performance of the CSSM-enss method is slightly better than that of the ISSM-ss method. However, the IEF-CSSM method is slightly more accurate because it uses an algorithm that does not require initial estimation values, but the final performance is weaker than that of the proposed method because of the poor decorrelation method.

### 4.2. Experimental Results

**Figure 5:**
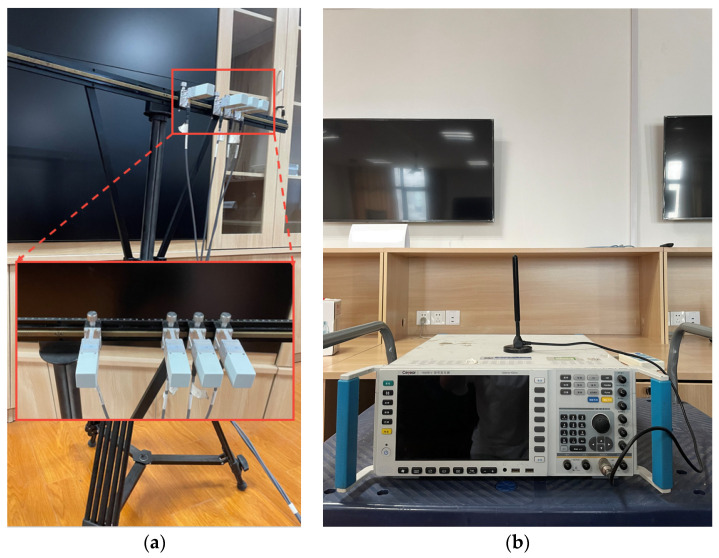
(**a**) is the sparse array used in the experiment, which used a tripod and a spirit level to ensure the stability of the support frame used for observation. (**b**) is the signal generator used in the experiment.

**Figure 6:**
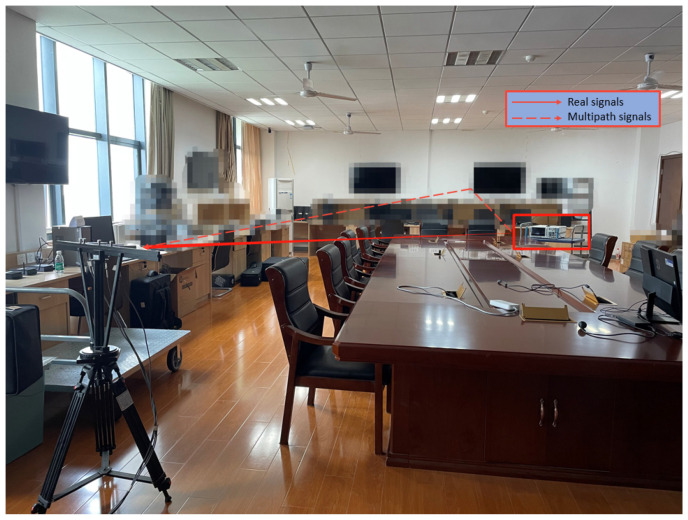
Experiment scene designed to satisfy the conditions required for coherent signals; we performed experiments inside of the room.

**Figure 7:**
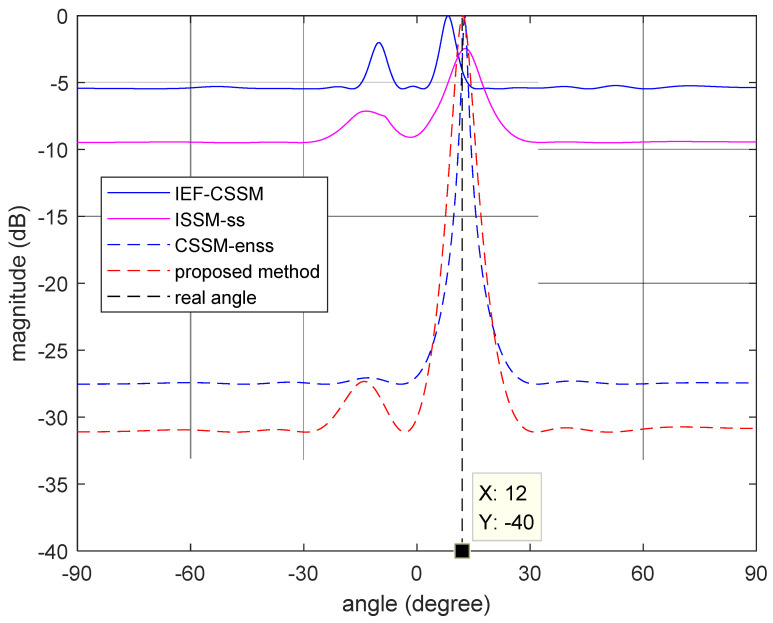
Spectrum of the proposed method for real data.

To demonstrate the effectiveness of the proposed method, we conducted a set of experiments inside of the room, in which a wideband signal with a bandwidth of 10 Mhz impinged on the sparse array from 12°. A total of 32 frequency bins were used for wideband DOA estimation, the experimental equipment and scene are shown in Figure 5 and Figure 6, respectively. As Figure 7 shows, the performance of the IEF-CSSM method in decorrelation is weaker than those of other methods in practical testing. On the other hand, the CSSM-enss method performs better than the ISSM-ss method but is worse than the proposed method. Furthermore, in Table 1, after the processing of the proposed method, there is a peak at 12.20° in the spectrum, while the real angle is 12°, which shows that the proposed method has satisfactory accuracy in practical application. And in order to ensure that the signal is coherent, we chose to perform experiments in an indoor environment, and the final processing results prove that the proposed method has the effect of decorrelation.

## 5. Conclusions

In this paper, we propose a DOA estimation algorithm for wideband coherent signals using the nested array. Firstly, a two-level nested array is used to ensure large DOF in the virtual array. Then, the focusing matrix is constructed without initial estimation to ensure that the initial estimation’s influence on the final estimation in the conventional algorithm is avoided. Furthermore, an enhanced spatial smoothing algorithm is used for decorrelation. Finally, the MUSIC algorithm is applied to perform high-accuracy DOA estimation. The proposed method has better decorrelation and higher accuracy than the ISMM-ss, CSMM-enss and IEF-CSSM methods. In our experimental tests, the proposed method is effective for coherent wideband signal processing, even in indoor environments, and we have demonstrated the advantage of the proposed method in relation to accuracy compared to other methods. In the future, we will investigate low-rank matrix recovery and denoising using the obtained covariance matrix before angle estimation, which could help to reduce residual interference on the covariance matrix.

## Figures and Tables

**Table 1:** Accuracy of the four methods for real data.

Method	DOA Estimation	Error Value
IEF-CSSM	8.25°	3.75°
ISSM-ss	12.80°	0.80°
CSSM-enss	12.70°	0.70°
Proposed method	12.20°	0.20°

## Data Availability

Not applicable.
